# Value pluralism supports portfolio approaches in conservation

**DOI:** 10.1007/s13280-026-02368-0

**Published:** 2026-03-08

**Authors:** Evelyn Brister, Yasha Rohwer, Michele Weber

**Affiliations:** 1 Philosophy, Rochester Institute of Technology, 92 Lomb Memorial Dr., Rochester, NY 14623 USA; 2 Humanities and Social Sciences, Oregon Institute of Technology, 27500 SW Parkway Ave, Wilsonville, OR 97070 USA; 3 Independent Researcher, 101 Strathmore Place, Los Gatos, CA 95032 USA

**Keywords:** Collaboration, Conservation planning, Environmental values, Inclusive decision-making, Portfolio approaches, Value pluralism

## Abstract

Portfolio approaches have gained support in conservation as they offer advantages similar to well-balanced investment portfolios. In this essay, we argue there are also ethical reasons to support portfolio approaches to conservation problems. First, we argue that value pluralism—the thesis that there are many diverse values, as opposed to a singular value—is the appropriate way to think about the value of ecosystems. We then argue that accepting value pluralism favors portfolio approaches to solving conservation problems because portfolios offer more chances to satisfy diverse values. Finally, we trace further implications for constructing conservation portfolios consistent with value pluralism. Specifically, portfolio planning should embrace inclusive decision-making, invest in diverse strategic assets, construct ladders of conservation solutions, and share knowledge and understanding widely to foster synergy across and between portfolios. A portfolio approach is more than a practical way to solve conservation problems: it is also ethically justified.

## Introduction

The overarching goals of conservation are to preserve, restore, and sustain functional and resilient ecosystems. Achieving conservation goals requires scaling up empirically based and socially supported conservation actions to preserve biodiversity and manage and restore ecosystems. Until recently, a broadly sketched “predict-and-prescribe” approach dominated the largely successful global conservation strategy (Schindler and Hilborn 2015; Langhammer et al. 2024). Under this broad umbrella, effective conservation action depends on accurate ecological forecasting followed by controlled interventions to address specific problems.

Many categories of conservation planning include a predictive component. These include analyzing big data to describe current threats (e.g., Wu et al. 2023; Sequeira et al. 2025), horizon scanning to systematically search for future threats (Sutherland and Woodruff 2009) and leveraging predictive modeling to identify patterns that may generate future conservation challenges (Travers et al. 2019). Following this predictive phase, different categories of conservation planning prescribe actions or interventions to tackle specific threats: for example, managing crown-of-thorns starfish outbreaks to protect coral from predation (Matthews et al. 2024) and developing cryopreservation techniques to preserve living genetic assets (Powell-Palm et al. 2023). We argue here that, as global change accelerates, conservationists need to be prepared with options beyond the predict-and-prescribe approach. We argue that ethical reasons favor portfolio approaches and examine ways that portfolio approaches are likely to enhance social support for conservation.

The predict-and-prescribe paradigm rests on the core assumption that scientific research can efficiently provide datasets that are sufficient for selecting a single best goal. However, this assumption is unrealistic in several ways. First, ecologists have shown that ecosystems are overwhelmingly complex and dynamic (Novak et al. 2011; Petchey et al. 2015), which entails that future states are never entirely predictable. Second, despite improvements in ecological models and forecasting, inadequate scientific information hinders prediction for many ecosystems (Schindler and Hilborn 2015). Third, the predict-and-prescribe approach assumes that relevant data can be collected on timescales that match funding availability and policy needs. Although policy processes can be slow, scientific research can be even slower, which is a challenge as ecological degradation and ecosystem change accelerate.

Granted, when the core assumption is met, then predict-and-prescribe approaches will have a solid evidential basis and scientific knowledge can help set the agenda for conservation. However, conservation also depends on values that underlie decisions about which conservation goals are most desirable or attainable, by whom, at what cost, and accompanied by what risks. Baumgaertner and Holthuijzen (2017) show that values inevitably influence the development of measures used in conservation science. Values play an increasingly important role as ecosystem changes become less predictable and ecosystem data is unavailable. When confronting these uncertainties, unless a decision-making process attends explicitly to values, more weight may be placed on values that have become incorporated into metrics but hidden from explicit evaluation (Shrader-Frechette and McCoy 1993). As a result, in addition to epistemic weaknesses, the predict-and-prescribe approach is more likely to obscure that values have been integrated into conservation science as well as what those values are.

Conservation goals should shift as new knowledge and different values are incorporated into conservation activities (Baumgaertner and Holthuijzen 2017), but the predict-and-prescribe approach rarely includes built-in structures to encourage this evolution. In contrast, when portfolio approaches force a constructive confrontation between the values that shape research choices and conservation planning, they are able to explicitly address both how and where values should be integrated into conservation science and when and where they should be updated as conditions change. This article explores how value-based considerations affect agenda-setting and why it is a benefit of portfolio approaches that they offer both more chances to make explicit the values that direct scientific research as well as more options to satisfy diverse values.

We enthusiastically acknowledge that predict-and-prescribe conservation benefits ecosystems and can be an important component of conservation portfolios. The landmark Langhammer et al. (2024) meta-analysis considered seven types of conservation action that are prescribed to target a predicted threat. Although most of the 186 studies in the meta-analysis document positive impacts, the interventions were singular and narrowly focused. When the ecosystem is tractable, this strategy is sufficient, as documented in the meta-analysis. We know that conservation outcomes improve when implemented early (Naujokaitis-Lewis et al. 2018), but that as conditions change, predicted threats must be reassessed. At many points in the process, conservationists practice judicious restraint (Brister et al. 2021), and as Schindler and Hilborn state, “forecasts narrowly limit uncertainties to those associated with a single potential outcome that is assumed to be predictable” (Schindler and Hilborn 2015, 953). Predictive analysis is laborious, and since it must precede action, it may not provide answers fast enough under current conditions of rapid change. In light of increasing urgency, complexity, and uncertainty, conservationists ought to be prepared to pivot from their original prediction and planned intervention to backup plans and new, emerging solutions. Portfolio approaches are frequently recommended because they can help manage risk under conditions of uncertainty and complexity.

Champions of portfolio approaches advocate for planning processes that incorporate flexibility and minimize risks while supporting ecosystem resilience (e.g., Ando and Mallory 2012; Aplet and McKinley 2017; Hobbs et al. 2017; Moore and Schindler 2022). Here, we argue that portfolio approaches are better at coping with persistent uncertainty about what the future holds and can protect the many ways that ecosystems—and the environment more generally—are morally valuable.

Conservation science is suffused with values (Soulé 1985), and conservationists recognize that the value of the environment is multifaceted. In philosophical ethics, the idea that there are many values that cannot be reduced to a single one is called *value pluralism* (Mason 2023). Once one accepts that there are many ways that the environment is valuable, one can also favor portfolio approaches for ethical reasons over more rigidly prescriptive approaches. Moreover, reflecting on the many ways that the environment is valuable can expand thinking about the value of a portfolio’s assets in ways that support strategic planning and inclusive decision-making.

Here’s our plan. We begin with a brief introduction to portfolio approaches. We then present reasons for accepting the thesis of value pluralism and how it applies to conservation. We draw out broader social implications of the use of portfolio approaches in conservation planning and argue that value pluralism endorses portfolio approaches not only for their ability to accommodate uncertainty and evolve following shifts in knowledge and values, but also for their capacity to embrace inclusive decision-making, to invest in diverse strategic assets that include tools and techniques, to ladder future conservation solutions, and to foster synergy across initiatives by sharing knowledge and resources. We end by showing how initiatives to restore American chestnut trees to the eastern US illustrate each of these points.

## Portfolio approaches: Assets, goals, and values

The aim of structuring conservation investments as portfolios is to incorporate more flexibility and resilience into conservation planning. Conservation portfolios acknowledge persistent uncertainty and can build in adaptability that accommodates new technologies, funding opportunities, social change, and shifts in value preferences and long-term goals. As with financial investing, portfolios provide multiple chances for success by anticipating change and distributing environmental and political risk.

In conservation, portfolios are constructed using various methods, ranging from broad incorporation of diversity into planning to strict selection of assets based on Harry Markowitz’s work on financial portfolios (Markowitz 1952). Markowitz’s Modern Portfolio Theory (MPT) argues that investors can design more efficient portfolios by maximizing returns for an acceptable level of risk. His insight transformed financial investing and formalized the idea that the collection is more important than the individual assets. For conservationists grappling with rapid change, MPT has been used to suggest efficient asset allocation early in the planning process. The following discussion of value pluralism covers portfolios in both broad and strict senses.

When assembling conservation portfolios, assets are typically taken to be genetic diversity or different geographical sites that include diverse biotic and abiotic components and provide ecosystem services. For instance, Crowe and Parker (2008) used MPT to guide reforestation planning by identifying allocations for the portfolio of white spruce seeds best adapted to the range of estimated future climatic conditions (a portfolio of genetic diversity). When Ando and Mallory (2012) applied MPT in the U.S. Prairie Pothole Region, they estimated habitat quality and independence as well as costs to protect three different geographic regions (a portfolio of diverse sites). Their method identified efficient land asset allocations to maximize future wetland habitat quality under four different climatic scenarios. In another prominent example of the application of MPT, the 50 Reefs project identified fifty reef localities globally that are less threatened by climate change and connected enough for larval dispersal (a portfolio of diverse sites that inherently also includes genetic diversity). They argued that protecting this portfolio of reefs would increase returns and maximize coral survival as sea surface temperatures continue to increase (Beyer et al. 2018). As these examples show, MPT can help conservation practitioners account for interdependence and maximize benefits for given risk tolerances (e.g., Mallory and Ando 2014; Eaton et al. 2019). Estimating and managing interdependent assets reduces the chance that the entire portfolio is impacted by extreme local events, such as locally invasive species, disease outbreaks, hurricanes, or forest fires (Schindler et al. 2010). Such applications of MPT can help funders, governments, and practitioners prioritize their efforts and efficiently allocate limited resources.

Although these efforts to spread risk and maximize conservation returns have provided valuable insight, it is important to acknowledge that applying MPT in conservation planning rests on value judgments, and that the underlying value judgments are often contested. First, MPT requires accepting a given level of risk and, therefore, accepting some possibility of loss. However, designating a given risk tolerance for a conservation portfolio is problematic because it requires placing a quantitative value on biodiversity and making explicit trade-offs that sacrifice assets. Second, in contrast to financial portfolios, where returns can be compared via a common currency, conservation success is defined differently by different stakeholders. As we will discuss in the next section, the value of conservation assets cannot be boiled down to a single currency; moral value is more complex than economic value.

Discussions about values—how they trade off and how they relate to broader social issues—are often missing from the discussion of portfolio approaches. We think this is a mistake because thinking about and acknowledging the many ways that the entities we aim to conserve are valuable provides strong support for portfolio approaches—but only if these diverse values are recognized. In the next section, we show how the thesis of value pluralism justifies portfolio approaches in conservation. Portfolio managers should be value pluralists.

## Value pluralism

Value pluralism is the thesis that multiple values may be correct, even though they may conflict with each other. ‘Value’ is what William James (1904) called a ‘double-barrelled’ term. It refers both to our *beliefs* about what is important (as when we talk about a person’s values) as well as to *what* has value (which might include people, biodiversity, integrity, beauty, etc.). Value monism is the position that there is one fundamental type of value, and that all other forms of value can be reduced to, or expressed as, this fundamental value. For instance, in utilitarian ethics, wellbeing or pleasure is the ultimate good. Value monism can provide a unitary solution to questions of value, allowing for decisiveness and clarity in resolving ethical questions. Value monism also provides a good conceptual fit with predict-and-prescribe approaches because when there is a single objective, it can more straightforwardly direct action. Here we argue instead for value pluralism, which is an uncomfortable fit with predict-and-prescribe approaches but is an easy fit with portfolio approaches. Value pluralism also makes sense of common intuitions about the complexity of environmental values.

In an influential 1985 paper, Michael Soulé called attention to the fact that normative commitments provide the ethical foundation for conservation biology. According to Soulé, the most important is “biotic diversity has intrinsic value, irrespective of its instrumental or utilitarian value” (Soulé 1985, 731). Something is intrinsically valuable if it is valuable for what it is (Korsgaard 1983). The belief that biodiversity is intrinsically valuable is expressed on the website of the Society for Conservation Biology, and the UN Convention on Biological Diversity affirms the intrinsic value of biodiversity. Conservationists have also expanded the circle of intrinsic value to include other properties of ecosystems. Complexity, integrity, autonomy, and beauty are a few examples of properties of systems that are said to have intrinsic value. Moreover, sentient creatures, genetic resources, and the biosphere as a whole have been given as examples of bearers of intrinsic value (Johnson 1991; Elliott 1997; Hettinger and Throop 1999).

While conservationists are often motivated to protect the environment by a belief in its intrinsic value, in practice they must often emphasize its economic or instrumental value to justify their actions to a wider public. Treating some environmental goods, such as ecosystem services, as instrumentally valuable does not negate—and may even depend on—underlying assessments of intrinsic value (Batavia and Nelson 2017). Entities may simultaneously possess multiple types of value. For instance, workers are instrumentally valuable for what they contribute to the economy, but they also possess intrinsic value as human beings. The environment is instrumentally valuable for human-specific ends—as sources of timber, pharmaceuticals, and food—and for the ends of non-human organisms. Proper relationships with the environment can also have value, as being in relationship with a particular landscape or non-human beings may partly constitute a cultural identity (Chan et al. 2016; Deplazes-Zemp 2024). And access to environmental goods, such as clean air and water, is essential for achieving environmental justice (Wenz 1988).

Given this extensive but not exhaustive list, it is difficult—and probably impossible—to boil down the myriad types of moral values in ecosystems to a single supervalue that would allow us to itemize and rank the value of specific systems and their properties. Another way to put this idea is that there is no common currency with respect to different types of intrinsic value; nor is there a common currency to measure the difference between intrinsic and instrumental value. Accepting that there are multiple irreducible values is called *value pluralism,* an important thesis in philosophical ethics (Mason 2023).

When the thesis of value pluralism is accepted, conflicts between diverse stakeholder values or between conflicting management objectives only need to be settled at a practical level, and not at a deeper conceptual level (Cook et al. 2023). Value pluralism accounts for the existence of trade-offs between different ways of prioritizing values. For example, a system could be very biodiverse yet have little integrity—think of a novel ecosystem that is full of introduced species. While the biodiversity of that system will be high, the integrity will be low, since the structure and function are novel and do not resemble the historical system that previously existed. A very controlled ecosystem, such as a garden, could be considered quite beautiful but could lack complexity and autonomy, since the components of the system interact only minimally and are continually human-controlled for aesthetic purposes. Preserving biodiversity and integrity may require removing—and sometimes killing—sentient creatures, especially nonnative predators (e.g., cats) or voracious, generalist herbivores (e.g., goats), as is often the case in island conservation. If the priorities placed on values must trade off, then it is not possible to reduce them to one common currency, or supervalue. The properties we value are distinct and, hence, to recognize them as contributing to the overall value of ecosystems requires a pluralist approach (O’Neill, et al. 2008).

Accepting value pluralism need not result in the breakdown of normative decision-making. Pluralism holds that many things have value; it does not hold that everything has value. Indeed, another virtue of the pluralist approach is that it allows decision-makers to be humble in the face of moral uncertainty and to recognize that different people value ecosystems for different reasons. Given the fallibility of humans, we could overlook properties of the environment that are valuable but that we have so far failed to recognize. Because this thesis holds that multiple values are worth pursuing—even when some trade off—a community may be justified in pursuing different goals via different means in different places at different times. Stakeholders and managers can and should be released from the obligation to settle the impossible question of which value—and consequently which conservation goal—is superior. Nor should they be expected to identify and prescribe a single strategy that satisfies all the preferred values. Accepting diverse and potentially conflicting values and selecting a portfolio of strategies co-designed with stakeholders can help conservation practitioners escape these impossible expectations and enable more democratic conservation decision-making processes (e.g., Bowie et al. 2020).

A pluralist position concerning environmental value supports portfolio approaches. For example, consider an idealized case covering a portfolio that tests two different treatments to ameliorate a conservation problem on two different site types. Because environmental properties can conflict or trade off, each site and each treatment may support some values while compromising others. If two sites conserves values A, B, C, and D, while the other two conserve values A, B, E, and F, then investing in both site types is the best way to conserve all of the ways that the environment is valued, and testing multiple treatments on the sites increases the odds of success. The predict-and-prescribe approach would select the most preferred treatment based on available scientific information and apply it to all sites, therefore sacrificing either values C and D or values E and F, while the portfolio approach aims to avoid the greatest loss of value.

Hobbs et al. (2017) show that the selection of sites to hold in a portfolio may incorporate a diversity of goals in order to maximize aggregate value. While one unit may be valuable because of its high integrity and high biodiversity, another unit that is in a degraded state may be worth including in a portfolio of sites for a different reason, not only because it offers to balance a specific risk but also because it offers other forms of value (such as recreational opportunities), is less costly to acquire and maintain, or has a high likelihood of regaining ecological function over a long enough timeframe. Given the difficulty of trying to pin down the many ways that ecosystems are valuable, and given that people value ecosystems for many different reasons, a portfolio approach encourages conservationists to cast a wide net when selecting and prioritizing conservation initiatives.

Some might think that a portfolio approach is just equivalent to unambitious conservation: conservation that is not taking the decisive, perhaps even risky, actions needed to help ecosystems. We don’t think this is the case. A portfolio approach doesn’t entail unambitious or sloppy conservation: as we will discuss later, such approaches require careful analysis, encourage deliberation, and can even involve cutting-edge genetic engineering techniques. In the following section, we trace out further implications of this emphasis on coordinating how value preferences are applied at multiple sites and in multi-stage planning.

## Expanding the scale and scope of conservation portfolios

The thesis of value pluralism removes the expectation that conservation activities aim for a single best goal. However, a common concern about value pluralism is that it entails relativism or an anarchic “anything goes” approach (Wolf 1992). But value pluralism says only that there is no supervalue that allows us to adjudicate disputes about values: it accepts the possibility that there are multiple valuable goods that may conflict or trade off (Mason 2023). Far from being anarchic, this thesis endorses deliberation and negotiation about how to design strategies that can evolve to serve multiple, potentially conflicting, values. In other words, value pluralism encourages mindful decision-making that incorporates diverse perspectives, acknowledges vested interests, and commits to revisiting decisions when knowledge, values, priorities, resources, and opportunities change over time.

In addition to providing an ethical reason for pursuing portfolio approaches for conservation planning, we argue that value pluralism guides best practices for preserving the variety of ways that functional, resilient ecosystems are valuable. These best practices include (1) reinforcing complex, inclusive decision-making; (2) greater appreciation for the potential benefits of diverse tools and techniques; (3) laddering conservation solutions as a strategy to accommodate parallel activities over extended development timelines; and (4) synergistic effects within and between conservation initiatives. Value pluralism brings conservation science into more active dialogue with communities, practitioners, funders, and supporters.

### Inclusive decision-making

Value pluralism acknowledges that things can be valuable in different and irreducible ways and implies that any of us may be unaware, fail to recognize, or change our minds about value propositions, especially as conditions change. Therefore, accepting value pluralism requires that communities identify and express their values, and it demands that deliberative processes support an exchange of reasons through dialogue. In the context of building conservation portfolios, value pluralism encourages attending to and learning from diverse views as we design environmental solutions (Norton 2015). Even when there seem to be intractable differences about what has value, a commitment to pluralism should motivate explicit processes of critical reflection and creative design in order to discover whether there exist ways to achieve results that support shared aims (Young 2000). Deliberation is both a form of inquiry and a path to political support, since “deliberation improves the quality and acceptability of collective decisions” (Saward 2003, 147). Although tensions, conflicts, and trade-offs between values may force pluralists into scenarios where there is no easy answer, deliberation serves the purpose of maintaining a rich diversity of critical ideas among conservationists (Matulis and Moyer 2017). Many conservation practitioners have experienced the struggle of conflicting values; acknowledging value pluralism can offer new language and help reduce discomfort with working through disagreement.

Recognizing diverse and irreducible values is an essential step toward creating social trust and building inclusive coalitions. Value pluralism is the philosophical commitment that stands behind openness to alternative value frameworks. Given the association between the causes of biodiversity loss and imperial value systems embedded in current legal norms and power systems, it is not surprising that positive ecological outcomes are correlated with the influence of indigenous people and their value systems (Dawson 2024). A commitment to value pluralism urges conservation planners to engage with indigenous and local communities.

Portfolios that support diverse values, such as those of indigenous and nonindigenous communities, can help to alleviate tension between people holding different priorities. Although it takes time and care to manage value conflict, there are a number of methods to structure conversations in ways that promote dialogue about deeply held values (e.g., Laursen et al. 2021), negotiate compromises, and expand the menu of options.

### Investing in tools and techniques: Strategic assets

Value pluralism supports conservation planning by expanding the evaluation of portfolio assets beyond sites and genetic diversity to include tools and techniques, or what we call *strategic assets*. As with standard portfolio assets, development of tools and techniques can be expensive investments that represent trade-offs and present differential risks. Various tools and techniques will often promote or protect different values; and so, employing a greater variety of tools and techniques can provide a better chance at preserving more of the ways that the environment is valuable.

Although many conservation programs successfully focus on a single vetted intervention strategy that is applied to a portfolio of sites, developing a suite of tools as strategic assets is another portfolio approach (e.g., Lahoz-Monfort and Magrath 2021). When conservation planners include multiple sites in their plan, they may also include a portfolio of strategic assets, testing experimental methods such as new management techniques or new restoration tools. For portfolios of genetic assets, strategic assets may include banking biomaterials, captive breeding, assisted reproduction, and reintroduction or translocation programs (e.g., Hobbs et al. 2017; Wilder et al. 2024). Allocating resources across diverse methods, tools, and techniques, including the development of new genetic tools and biotechnologies (Corlett 2017; Piaggio et al. 2017), can help set up many shots on goal.

A survey of the many tools and techniques being developed by diverse coral reef conservation initiatives demonstrates how tools and techniques might be understood as strategic assets. Coral gardening maintains living coral reefs in the wild (Rinkevich 2014). At the same time, advanced aquarium husbandry improves the survival of living colonies in captivity, maintaining as genomic assets the corals themselves as well as associated taxa (National Academies 2019). Cryopreservation tools and techniques ensure access to living coral gametes, tissues, and symbionts that can buttress other efforts even if they go extinct in the wild and captivity (Powell-Palm et al. 2023). Assisted reproduction informed by genetic and genomic data can expand temperature tolerance in corals and symbionts (Hagedorn et al. 2021; Lamb et al. 2024). Over longer timeframes, gene editing techniques might be developed for corals and their symbionts that will enable future scientists to experiment with specific functional changes (Cleves et al. 2018, 2020).

The different tools and techniques listed here do not all align with the same sets of values. For example, genetically modifying coral may help preserve biodiversity and wildness but could be construed as compromising the integrity of a system or species if foreign DNA is used in the modification. Viewing these unconsolidated efforts holistically, as *society’s* conservation portfolio, could further enable rapid experimentation, efficient feedback loops, and coordinated action. One way value pluralism enhances portfolio approaches is by helping connect the dots between various values and how different tools can further those values. This pluralistic perspective encourages the development of new tools and techniques to support additional values as well as the exchange of ideas and tools between initiatives with shared values. The next two sections highlight additional advantages of investing in portfolios of strategic assets.

### Laddered conservation solutions

Portfolios of traditional and strategic assets generate opportunities to ladder conservation solutions. Judd et al. (2011) showed that laddering bonds with variable maturities is part of building optimal financial portfolios, and now we assert that conservation technologies can also be laddered to accommodate variable development timelines and diverse values. Conservationists know that avoiding delay improves outcomes (Naujokaitis-Lewis et al. 2018; Brister et al. 2021) but few conservation solutions can be deployed immediately. Although new technologies are emerging faster than ever, most solutions for conservation require substantial development prior to field-testing and eventual implementation (Berger-Tal and Lahoz-Monfort 2018; Ockendon et al. 2021; Schultz et al. 2023; van Oosterhout et al. 2025). For example, land preservation strategies require complex negotiations with landowners and sometimes land sales or trades (Brugnach et al. 2017; Capano et al. 2019). Policy solutions require political will, and integrating co-design solutions can be slow (Little et al. 2024). When considering conservation of a new taxon, captive breeding programs must hire appropriate reproduction and husbandry experts and construct large-scale facilities before collecting individuals and running the appropriate genetic testing to optimally pair them (Comizzoli and Holt 2019). By taking a laddered approach, practitioners anticipate development timelines and build a plan that stacks solutions to become usable at strategic times. Laddering also ensures that short-term goals can be achieved while long-term assets are under development.

Designing portfolios to try new ideas as they mature makes sense in a complex, dynamic world. Organizing a multi-solution strategy can be fraught, and although a ladder will not avoid difficult trade-offs, realistic development timelines may help provide structure and context. A ladder of solutions provides the opportunity to learn as you go. Monitoring the results of early experiments will generate insights that inform decisions about ideas with longer time horizons, potentially improving subsequent projects (Stier et al. 2022). Some compelling solutions require new technologies; however, developing new technologies is slow and incremental. By laddering more immediate solutions under complex long-term development trajectories, practitioners can hedge against unavoidable delays in the lab. And since earlier low-risk solutions are likely to improve the situation to some degree (Langhammer et al. 2024), the potential failure inherent in high-risk, high-reward ideas may feel more acceptable. By implementing less technical solutions concurrently with long-term technology development, fundraisers can build on incremental success to generate financial support for later stages of the work.

Portfolios of strategic assets put eggs in more than one basket, and a laddered portfolio structures the decisions about when to invest in which basket. Although predict-and-prescribe approaches continue to generate success—and it is tempting to optimize for a silver bullet solution—a laddered portfolio builds in opportunities for incremental progress and makes moonshots less daunting. Ladders make it explicit that not everything will happen simultaneously. They build in a premise of time and patience, helping to accommodate diverse timelines and perspectives and welcome more values into the big tent. Although the results may be less instantaneous and, therefore, less dramatic for stakeholders and funders, ladders lessen the pressure on practitioners to get it right the first time with a big, expensive solution. Strategically laddering solutions and implementing different solutions as they mature can help slow-but-steady conservationists win the race against environmental decline.

### The potential for synergy

One obvious advantage of portfolios is that synergies may emerge when multiple conservation efforts contribute to a larger goal, both within a portfolio of assets and across independent initiatives. In some conservation contexts, practitioners follow the traditional model of financial portfolios by aiming for independence among portfolio assets, so that stochastic events do not compromise the effort. However, when conservation assets include sites or genetic resources within a region, they are likely to share risk factors. For example, coral reefs experience global- and regional-scale threats such as warming sea surface temperatures, ocean acidification, coastal pollution, and destructive fishing practices.

However, conservation assets are often linked in positive ways that outweigh the risks and do not have a direct analogy in financial portfolios. Since biodiversity conservation is a global and ethically motivated mission, successful conservation can benefit parallel projects and increase synergy. For instance, building corridors may increase value for low-quality sites and produce refugia. Simultaneously, corridors can create new source populations that disperse further in response to changing ecological conditions. Investing in the development of new tools and techniques for more impactful direct ecosystem interventions can benefit multiple conservation projects, reducing risk and improving outcomes, especially when knowledge-sharing is prioritized.

Although this paper focuses on building a portfolio of direct ecosystem-oriented actions, from a broader perspective, operational strategies that encourage positive behavior could synergistically contribute to the same conservation goals by raising additional funding or generating stable management. For example, public–private partnerships are consolidating financial resources to address large-scale conservation challenges like protecting flyways for migratory birds (USFWS). In addition, the UN is exploring new mechanisms to generate funding for conservation at the international scale, such as the Global Fund for Coral Reefs (GFCR). In addition, payment for ecosystem services or payment by results strategies can be used to monetarily incentivize conservation action (Gibbons et al. 2011; McDonald et al. 2018). And finally, long-term conservation agreements such as land trusts or conservation covenants can help ensure that operations can make long-term conservation and restoration management plans (Capano et al. 2019; Richardson et al. 2024). Developing new operational strategies can synergistically fund and further the conservation impact of direct ecosystem interventions.

As in the global effort to conserve coral reefs described above, multiple dispersed strategies may improve outcomes for the same conservation target. Conservation is challenging because there are many unknowns and many lessons to learn. Acknowledging value pluralism against this background of contingency and fallibility can nurture a culture of resource- and knowledge-sharing. Practitioners often rightly acknowledge that conservation assets are interconnected and that collaboration across a portfolio or with outside initiatives can reinforce all initiatives.

### Example of a portfolio underway

American chestnut restoration illustrates how value pluralism can be a basis for positive social impact and effective restoration. Like many conservation initiatives, American chestnut restoration involves partnerships between government agencies, non-profits, academic researchers, and businesses. The American Chestnut Foundation (TACF), the United States Department of Agriculture, university researchers, and other partners, including privately owned tree nurseries, have crafted concurrent complementary strategies over many years. They have created synergy between initiatives that are independently controlled yet in certain ways interdependent. TACF and its partners have persevered through difficult deliberations about strategy because their various activities are associated with different ways to interpret, apply, and prioritize the ethical values associated with native, hybrid, and genetically modified varieties. These activities have included (1) the identification, collection, seed storage, and potential cultivation of native American chestnut trees (a native tree preservation strategy; Sandercock et al. 2024), (2) backcross breeding between American and Chinese species (a hybridization strategy; Clark et al. 2023), and (3) the creation of a transgenic blight-tolerant variety (a genetic modification strategy; Newhouse et al. 2014; Newhouse and Powell 2021). In addition, partners have developed and shared cultivation techniques to speed reproduction, facilitate growth, and reduce the damage of blight infections, and TACF has pursued research on biocontrol, a strategy to lessen the virulence of chestnut blight (TACF 2016). Each strategy has required significant investment of labor and financial resources, and they have different estimated timelines for maturity.

In 2016, TACF’s science committee adopted a “3BUR Proposal for Integrated Research.” With regard to value pluralism, when tolerance for diverse values among chestnut enthusiasts was cultivated, the independently managed initiatives built a culture of knowledge-sharing and collaboration that raised public support for chestnut restoration. We do not yet know which chestnut restoration strategies will be most successful or how long they will take to mature, but the simultaneous pursuit of multiple strategies increases the chance of success. They stand to benefit each other and can be deployed as a ladder as they become ready. Even though heritable blight resistance has not been documented among native trees, maintaining orchards of legacy trees is a critical source of genetic diversity for the other strategies. It may yet turn out that both hybridized and genetically modified varieties will have a role in future restoration scenarios, and both depend on incorporating locally adapted phenotypes into seed stock. All three reproductive strategies have benefited from strategic investments in new techniques that improve success rates for germination, cultivation, reproduction, and defense against infection.

Synergy between strategies in a portfolio is especially important when we remember that different strategies will support different values. Value pluralism can help fortify fragile coalitions. As coalitions are established, stakeholders may struggle to build trust because they fear conflicting priorities. Acknowledging and embracing value pluralism encourages expecting and accepting the conflicting priorities that emerge when diverse values come together. In the case of American chestnut restoration, some supporters oppose genetic modification because they believe it aligns with a paradigm of increased technological intervention (Diehm 2023). Others are opposed to hybridization because it may compromise genetic integrity or have unintended ecological effects. Yet, others are open to any strategy that will result in chestnut restoration that can be sustained. At the moment, collaboration among these initiatives has broken down, in part because of value differences and disagreements over strategic priorities for implementing restoration efforts. Some supporters are concerned that such differences will hinder successful restoration (Beehler 2025).

Committing to value pluralism welcomes the ensuing fraught-but-necessary negotiations, underscoring that they are an important part of coalition-building. As the environmental crisis accelerates, we need all hands on deck. Coalitions built on a platform of value pluralism will be stronger and more synergistic. A portfolio-oriented approach, established concurrently with practices that acknowledge value pluralism, will support more successful coalition-building and encourage the culture of synergistic knowledge and resource-sharing that we need to achieve big conservation goals. As coalitions grow and diversify, the resource pool will expand as new funders jump in to support the values that resonate with them.

## Why now?

We have argued that not only is value pluralism an appropriate way to think about the value of ecosystems, but also that a commitment to value pluralism favors portfolio approaches to conservation. We have also argued that value pluralism encourages portfolio approaches for their predisposition to embrace inclusive decision-making, invest in diverse strategic assets, ladder potential conservation solutions, and share knowledge and understanding widely to foster synergy across and between portfolios. Conservationists have many prudential reasons to pursue portfolio approaches; value pluralism adds a normative justification that permits them to be more deliberate and self-aware of the societal and ethical implications of portfolio design.

Portfolio approaches encourage anticipatory planning at multiple spatial and temporal scales. They provide structure to manage risk within and among management units, especially where there is an intent to manage for multiple value preferences and where the socio-ecological context is undergoing change (Magness et al. 2022). The diverse assets and strategies woven into a portfolio encourage practitioners to experiment with new ideas. As monitoring and evaluation techniques have improved and matured, even complex and diverse portfolios of conservation assets are now more tractable. The longer timeframe made explicit in portfolio approaches acknowledges that measurement and evaluation should be customized and that the data collected will help strategies evolve accordingly.

We might also ask why support is building around portfolio approaches *now*, following concerns expressed about managing for single species and other predict-and-prescribe approaches in the 2000s (Lindenmayer et al. 2007; Botrill et al. 2008; Carwardine et al. 2009; Schindler and Hilborn 2015). As information technologies have improved, data can be shared more efficiently, and communication and coordination within and across projects is easier. Remote sensing and other data collection technologies are getting easier, faster, and cheaper. The accumulation of high-quality data and increasing computational power and literacy will continue to improve quantitative modeling and forecasting methodologies and to supercharge increasingly sophisticated analysis techniques (Ball-Damerow et al. 2019; Cazalis et al. 2022). Machine learning approaches and other big data strategies will make even bigger leaps forward following the growing investment and development of artificial intelligence technologies, further enabling the more complex and shifting balance that portfolio management requires.

Another reason that portfolios are becoming common is that collaboration rather than competition is now recognized as an effective response to resource scarcity in the conservation sector. Lindenmayer et al. (2007) recommend employing multiple complementary strategies but note that funding, time, and expertise are scarce and, as a result, conservation planners tend to prioritize one or a small number of strategies. We believe that portfolio approaches afford an option for various coordination arrangements, including public–private partnerships. Value pluralism also suggests opportunities to broaden public support for government-funded conservation initiatives and create new sources of philanthropic support for private ones (Gruby et al. 2023).

In a time of increased urgency to address biodiversity loss, there is an ethical imperative to preserve diverse forms of environmental value for the future. Functional, resilient, complex, and biologically rich ecosystems are the legacy we want to leave. Acknowledging value pluralism is a crucial part of building portfolios that meet our most ambitious conservation goals.

## Data Availability

This is a theory paper and does not rely on data other than the referenced texts.

## Declarations

### AI Statement

We did not use AI in the production of this manuscript.
